# Brain network centrality following stress in adults with major depressive disorder and childhood trauma

**DOI:** 10.1016/j.ynstr.2026.100817

**Published:** 2026-04-12

**Authors:** C. Broeder, J.M. Pasteuning, T.A.A. Broeders, F. Linsen, M.M. Schoonheim, M.S.C. Sep, C.H. Vinkers

**Affiliations:** a Amsterdam UMC Location Vrije Universiteit Amsterdam, Department of Psychiatry, Boelelaan, Amsterdam, 1117, the Netherlands; b Amsterdam UMC Location Vrije Universiteit Amsterdam, Department of Anatomy & Neurosciences, Boelelaan, Amsterdam, 1117, the Netherlands; c Amsterdam Neuroscience, Mood, Anxiety, Psychosis, Sleep & Stress Program, Amsterdam, the Netherlands; d Amsterdam Public Health, Mental Health Program, Amsterdam, the Netherlands; e GGZ InGeest Mental Health Care, Amsterdam, the Netherlands

**Keywords:** Major depressive disorder, Childhood trauma, Acute stress, Recovery, Trier social stress test, Resting-state fMRI, Network centrality

## Abstract

**Background:**

Childhood trauma (CT) is a major risk factor for major depressive disorder (MDD), potentially via altered stress system development. Previous studies have shown stress-related changes in brain network function in clinical populations, but evidence in MDD with CT remains scarce. This study examined changes in functional brain networks in adults with MDD and CT during acute and delayed phases following stress.

**Methods:**

Resting-state functional magnetic resonance imaging (fMRI) was acquired during acute (15 min) and delayed (135 min) stress phases following the Trier Social Stress Test in 66 adults with MDD and CT and 33 controls. Voxel-wise eigenvector centrality (EC) mapping quantified the network importance of eight functional brain networks. Subjective stress and affect were measured using visual analog scales.

**Results:**

Both groups showed significant subjective stress responses, with greater increases in tension in the MDD + CT group; affective reactivity did not differ. No significant changes in EC were observed between acute and delayed phases in any network, nor were there main effects of group or group × time interactions. Sensitivity analyses in severe MDD and multiple CT subtypes confirmed these null findings.

**Conclusions:**

Network centrality did not differentiate individuals with MDD and CT from controls following stress, while subjective tension responses were higher in the MDD + CT group. These results suggest that global resting-state network centrality may not be a sensitive indicator of stress vulnerability following CT. Future multimodal studies incorporating task-based paradigms and biological markers are warranted to elucidate the neural and behavioral pathways linking MDD, CT, and stress reactivity.

## 1 Introduction

Major depressive disorder (MDD) is a highly prevalent and debilitating psychiatric disorder (Malhi et al., 2018; Marx et al., 2023), ranking among the leading causes of disability and mortality worldwide (World Health Organization, 2020; Kessler et al., 2013). Childhood trauma (CT), defined as emotional, physical or sexual abuse or emotional or physical neglect before the age of 18 (McKay et al., 2021; Kuzminskaite et al., 2022a) is a well-established risk factor for both the onset and a poorer course of MDD (McKay et al., 2021; Kuzminskaite et al., 2022a). Approximately 46% of adults with MDD report a history of CT (Nelson et al., 2017), with even higher rates among those with persistent depression (Struck et al., 2020). CT is associated with earlier MDD onset, greater symptom severity and persistence (Teicher et al., 2013; Nanni et al., 2012; Kuzminskaite et al., 2022b), and increased anxiety, suicidality, insomnia, and long-term functional impairment (Hovens et al., 2012; Miniati et al., 2010). Among individuals with MDD, CT is linked to widespread alterations in resting-state functional connectivity including reduced functional connectivity and altered brain network properties within the prefrontal-limbic-thalamic-cerebellar circuit (Wang et al., 2014; Shin et al., 2025), increased functional connectivity within the default mode network (Lin et al., 2024), and altered within-network connectivity of the dorsal attention network and subcortical regions and between-network connectivity involving task-positive and sensory networks (Yu et al., 2019). Furthermore, recent neuroimaging studies highlight a complex interplay between CT and MDD, including both distinct and cumulative alterations in resting-state connectivity across regions and networks involved in emotion regulation, sensory processing, reward, and cognitive control (Luo et al., 2022; Zou et al., 2024; Xia et al., 2024). However, prior research on CT has largely focused on fronto-limbic circuits (Ross et al., 2021), while studies on whole-brain resting-state functional connectivity are scarce.

The long-lasting vulnerability linked to CT may result from alterations in stress system development during sensitive periods of neuroplasticity (Murphy et al., 2022; Hakamata et al., 2022; Pasteuning et al., 2025a; Engel et al., 2020). Prolonged cortisol exposure and glucocorticoid receptor (GR) overactivation during these windows can interfere with maturation of the hypothalamic-pituitary-adrenal (HPA) axis (Lupien et al., 2009; Roberts et al., 2019; Van Bodegom et al., 2017), with lasting effects on stress reactivity (Gray et al., 2017). Accordingly, CT has been linked to persistent alterations in cortisol reactivity to stress (Murphy et al., 2022; Bunea et al., 2017) and neural processing of threat, reward, and emotion (Hakamata et al., 2022; Pasteuning et al., 2025a; Teicher et al., 2016; McCrory et al., 2017), even in the absence of overt psychopathology (Pasteuning et al., 2025a; McCrory et al., 2017). Although these adaptations may enhance immediate threat detection, they can impair long-term stress regulation. In particular, insufficient deactivation of the stress response may prolong catecholaminergic activity, increasing chronic stress and vulnerability to psychopathology like MDD (Van Bodegom et al., 2017). As such, the impact of CT may be most evident under stress, making stress responses a critical target for understanding the etiology of MDD with CT.

An adequate stress response requires both rapid reactivity and subsequent recovery. These processes rely on a dynamic reallocation of neural resources, marked by temporally specific reconfigurations of large-scale brain networks (Joels et al., 2012; Joels et al., 2009; Hermans et al., 2014; de et al., 2005; Ulrich-Lai et al., 2009). During acute stress, connectivity of the ventral attention network (VAN), involved in detecting salient stimuli, increases. This is often accompanied by context-dependent alterations in the frontoparietal network (FPN; executive functions), and default-mode network (DMN; self-referential processing) (Hermans et al., 2014; Zhang et al., 2019; Van et al., 2017b). During recovery, changes in VAN and FPN connectivity roughly normalize (Hermans et al., 2014), while the dorsal attention network (DAN), involved in guiding directed attention, becomes more central (Broeders et al., 2021). This dynamic shift supports vigilance and adaptive responding under acute stress and restores attentional control and higher-order cognition during recovery (Broeders et al., 2021). Disruptions in neural recovery have been linked to psychopathology: genetically at-risk siblings of individuals with schizophrenia failed to downregulate VAN connectivity and showed atypical DMN and reward-related activity (Van et al., 2018; Van der Stouwe et al., 2019 van Leeuwen et al., 2019), and patients with bipolar disorder did not show the expected increase in DAN centrality (Broeders et al., 2021). However, data on changes in functional brain connectivity over time following stress induction in individuals with MDD and CT are lacking. Understanding these stress-response trajectories might be key to identifying neurobiological mechanisms of stress vulnerability in this high-burden group.

This study aimed to examine stress-response network dynamics in individuals with MDD and CT by assessing changes in network centrality based on resting-state functional magnetic resonance imaging (fMRI) scans during acute (±15 min) and delayed (±135 min) stress phases following a standardized stress induction paradigm (Trier Social Stress Test; TSST) compared to healthy individuals without CT. Baseline data from the ‘*REStoring mood after Early life Trauma - medication*’ *(RESET-medication)* and ‘*stRess systEm dynAmics in Childhood Trauma-related depression’* (*REACT*) studies were analyzed (Linsen et al., 2023). These studies followed identical protocols, allowing us to investigate changes in brain network connectivity following stress in a relatively large, well-characterized cohort. By focusing on this high-burden group with MDD and CT, we provide an initial investigation into potential impairments in neural trajectories following stress. Because of the widespread impact of stress on functional brain networks, we conducted a whole-brain, data-driven network centrality analysis to capture potential group differences in neural stress processing. Given that an adaptive stress response relies on dynamic switching between functional brain networks (Broeders et al., 2021; Van et al., 2018; van et al., 2019), network centrality provides a suitable measure, as it allows capturing the extent to which specific networks become more or less influential within the brain during stress. In addition, because centrality measures do not rely on a priori assumptions (Lohmann et al., 2010), they are particularly suitable for this initial investigation. In line with previous work, we expected increased VAN centrality during the acute phase of the stress response, as well as increased DAN and decreased VAN centrality during the delayed phase, potentially accompanied by changes in FPN and DMN centrality in either phase. Based on earlier work in other clinical and at-risk populations, we hypothesized that these network shifts during the delayed phase would be diminished in individuals with MDD and CT compared to controls.

## 2 Methods

### 2.1 Participants

This study included 66 adults with moderate to severe MDD and CT that participated in the baseline measurement of the RESET-medication study, a randomized controlled trial examining the impact of glucocorticoid receptor antagonism on depressive symptom severity (Linsen et al., 2023). In addition, 33 healthy controls without MDD and CT from the REACT study were included. Both studies used an identical baseline protocol and were conducted at the same research site. CT was defined as scoring above the validated cut-off score for at least one domain of the Childhood Trauma Questionnaire – Short Form (CTQ-SF) (Bernstein et al., 1994): physical neglect ≥10, emotional neglect ≥15, sexual abuse ≥8, physical abuse ≥10; emotional abuse ≥13. MDD diagnosis was confirmed or ruled out at baseline using the Mini International Neuropsychiatric Interview-Simplified (MINI-S) interview (Overbeek et al., 2019). Upon participation, all depressed individuals scored ≥26 on the Inventory of Depressive Symptomatology-Self Rated (IDS-SR), which classifies as moderate to severe depression (Rush et al., 2000). Exclusion criteria for all participants included a primary diagnosis of post-traumatic stress disorder or acute stress disorder, a lifetime diagnosis of borderline personality disorder, bipolar disorder, or any psychotic disorder, as well as current substance abuse, chronic adrenal insufficiency, neurological disorders, and MRI contraindications. Given the nature of the RESET-medication trial, individuals with MDD and CT were also excluded if they started depression treatment in the week before or after the start of the intervention or used drugs being CYP3A4 inhibitors, inductors or substrates, CYP2C8 or CYP2C9 substrates, glucocorticoid antagonists or systemic corticosteroids. For females participating in this trial, additional exclusion criteria were a history of unexplained vaginal bleeding or endometrial changes as well as pregnancy and breastfeeding. For healthy controls, additional exclusion criteria were past or current psychiatric disorders and a history of CT, as measured with the CTQ-SF. Both studies were approved by the medical ethical committee of the Amsterdam University Medical Center and all participants provided written informed consent.

### 2.2 Study design and stress induction

All data analyzed here were collected following the baseline protocol described previously (Linsen et al., 2023), and all (f)MRI data were acquired on the same scanner. Neither individuals with MDD and CT nor controls had been subjected to any (medical) intervention at the time of scanning. Resting-state scans were acquired during an acute and delayed stress phase, at approximately 15 and 135 min relative to the onset of stress induction, respectively. These resting-state scans were part of a scan protocol with a total duration of 45 min. In between scanning sessions, participants had a 60-min break. The timing of the acute and delayed scanning sessions was selected based on prior literature describing stress response patterns of cortisol and aim to reflect stress reactivity and recovery (de et al., 2005; Zhang et al., 2019). Stress was induced in both groups using the Trier Social Stress Test (TSST), a well-established paradigm for eliciting psychosocial stress (Kirschbaum et al., 1993). The TSST consists of a 3-min anticipation period, followed by a free speech and a mental arithmetic task performed in front of an audience instructed to withhold positive feedback. The total duration of the TSST is approximately 15 min. To assess stressor impact on subjective experience, affect (positive vs. negative) and tension (tense vs. relaxed) were measured using a 10-point Visual Analog Scale (VAS) at −5, +8, +30, +75, +135, +150, and +195 min relative to the start of the TSST.

### 2.3 MRI acquisition

Data were acquired on a Philips Ingenia 3.0T scanner (Philips Healthcare, Best, The Netherlands) using a 32-channel head coil. A 3D T1-weighted anatomical scan was acquired for registration purposes and resting-state fMRI sans were acquired using T2∗-weighted echo planar images (EPI). Scan parameters were as follows: repetition time (TR) = 2000 ms; echo time (TE) = 27.63 ms; resolution = 3 × 3 mm; field of view 240 × 240 mm; 37 sequential slices; flip angle (FA) = 76°; 297 volumes (duration of 10 min). For resting-state scans, participants were instructed to lie still, let their thoughts wander, and keep their eyes open.

### 2.4 MRI preprocessing

Images were visually inspected for incomplete coverage and artefacts. Preprocessing was performed using a validated preprocessing pipeline optimized for resting-state fMRI data (Broeders et al., 2024), implemented using Freesurfer (Fischl, 2012) and FMRIB Software Library (FSL) version 6.0.7.6 (http://www.fmrib.ox.ac.uk/fsl). In brief, the pipeline included distortion correction (SynBOLD-DisCo) and brain extraction (HD-BET (Isensee et al., 2019)), followed by motion correction, slice-time correction, non-linear registration to standard space (MNI-152), and Gaussian smoothing (5 mm isotropic kernel), all carried out using FSL's FEAT (Woolrich et al., 2001). Additional steps included motion artefact removal using ICA-AROMA (Pruim et al., 2015), residual nuisance regression (i.e., mean white matter and ventricle signal, motion parameters, and the derivatives of each), and high-pass temporal filtering (100 s). Finally, images were transformed and resampled to 4 mm standard space.

### 2.5 Network centrality

Network centrality was primarily assessed using eigenvector centrality (EC), a graph-theoretic measure that quantifies how well a node is connected within the overall network structure by considering both its direct connections and the extent to which its neighboring nodes are connected within the network (i.e., their centrality) (Lohmann et al., 2010; Wink et al., 2012). By assigning higher EC values to voxels connected to other highly connected voxels, EC mapping provides a voxel-based, data-driven approach that captures the importance of a node within a network. This allows for the examination of potential brain network reconfiguration during the stress response (Lohmann et al., 2010). Voxel-wise EC mapping was performed for each participant using fastECM (Wink et al., 2012). As individual graph measures may only capture specific aspects of the functional connectome, combining multiple metrics is generally recommended (Mullin et al., 2025; Zuo et al., 2012). Therefore, we complemented EC with degree centrality (DC), a simpler centrality measure that reflects the number of a node's direct connections within a network without taking into account the connections to its neighboring nodes (Lohmann et al., 2010). With that, DC represents a more local measure of network importance. Resting-state networks were defined using the seven-network cortical parcellation atlas by Yeo et al. (2011) (Yeo et al., 2011), with an additional deep gray matter (DGM) network based on segmentations obtained using FSL's FIRST. The parcellation combined 14 FIRST-based DGM regions with 210 cortical regions from the Brainnetome atlas (Fan et al., 2016). Resting-state networks were mapped onto the regional atlas, and regions were matched to the network they overlapped with most. This resulted in the analysis of eight networks: the DMN (46 regions), FPN (24 regions), VAN (23 regions), DAN (27 regions), visual (VIS) network (30 regions), somatomotor network (SMN; 33 regions), limbic (LIM) network (27 regions) and DGM network (14 regions). The DGM network contained only voxels that were present in at least 50% of all FIRST segmentations and were attributed to DGM. Centrality was averaged across all voxels within a network.

### 2.6 Statistical analysis

All statistical analyses were performed using R version 4.4.3 (R Core Team, 2016). Group differences in demographic characteristics were examined using independent-samples t-tests for continuous variables and chi-square tests for categorical variables. To confirm successful subjective stress induction, VAS ratings for affect and tension were compared between baseline and peak timepoints within groups using paired t-tests. Baseline was defined as the first timepoint (t = −5 min; before stress induction), and peak as the highest rating observed at either the second (t = +8 min; during stress induction) or third (t = +30 min; immediately after stress induction) timepoint. Independent-samples t-tests were then used to examine whether these changes from baseline to peak differed between groups. In addition, area under the curve with respect to ground (AUCg) and with respect to increase (AUCi) were calculated for both VAS measures across timepoints −5 to 30 min (i.e., from before to immediately after the TSST), following the approach described by Khoury et al. (2015) for cortisol summary indicators (Khoury et al., 2015). These timepoints were selected in accordance with previous work showing that VAS scales on perceived stress were significantly elevated during the TSST and decreased rapidly thereafter (Hellhammer et al., 2012), making timepoints beyond 30 min less informative for assessing the subjective stress response. Group differences in AUCg and AUCi were likewise evaluated using independent-samples t-tests.

Changes in network centrality (EC and DC) were first assessed within each group using linear mixed models (LMMs) to test for a main effect of time (acute vs. delayed stress phase). Subsequently, 2x2 LMMs, including group (MDD + CT vs. healthy controls) as a between-subject factor and time (acute vs. delayed stress phase) as a within-subject factor, were used to evaluate main effects of group and time, as well as their interaction. A significant group × time interaction would indicate that trajectories of network centrality following stress differ between groups. All models were adjusted for age (mean-centered) and sex. Moreover, sensitivity analyses for EC models were conducted by repeating the models in two subsets of the MDD + CT group: participants with severe depressive symptoms (IDS-SR score ≥39 (Rush et al., 2000) and, separately, those reporting two or more CT subtypes. Analyses were performed for each of the eight resting-state networks. All analyses, including subjective experience measures and fMRI network outcomes, were corrected for multiple comparisons using false discovery rate (FDR)-adjusted p-values calculated with p.adjust; values below 0.05 were considered statistically significant.

## 3 Results

### 3.1 Participant characteristics

Of the 66 participants in the MDD with CT group, one was excluded due to missing fMRI data during the delayed stress phase, and one was excluded due to structural brain abnormalities that could affect the interpretation of fMRI data. This resulted in a final sample of 64 participants with MDD and CT (44 females; mean age = 45.5 ± 12.2 years) and 33 controls (10 females; mean age = 55.0 ± 12.0). The MDD with CT group was significantly younger and included a higher proportion of females and a lower proportion of individuals who obtained higher education than the control group (all *p* < 0.05). Demographic and clinical characteristics of the study sample are displayed in Table 1.

**Table 1:** Demographic and clinical characteristics of study sample.

Characteristics	MDD with CT (*n* = 64)	Controls (*n* = 33)	Test statistic	*p*-value
Age (years), Mean ± SD	45.5 ± 12.2	55.0 ± 12.0	t = −3.63	**<0.00**1^a^
Sex (male/female), No. (%)	20/44 (31.2/68.8%)	23/10 (69.7/30.3%)	X^2^ = 12.7	**<0.00**1^a^
Higher educational level^a^, %	48.4%	72.7%	X^2^ = 4.3	**0.0**4^a^
Depression severity (IDS-SR), Mean ± SD	39.4 ± 7.9	3.5 ± 2.9	-	-
Childhood trauma severity (CTQ total), Mean ± SD	60.3 ± 12.1	29.8 ± 4.2	-	-
Emotional neglect, No. (%)	55 (85.9%)	-	-	-
Emotional abuse, No. (%)	46 (71.9%)	-	-	-
Physical neglect, No. (%)	30 (46.9%)	-	-	-
Sexual abuse, No. (%)	23 (35.9%)	-	-	-
Physical abuse, No. (%)	17 (26.6%)	-	-	-
Two or more CT subtypes, No. (%)	52 (81.3%)	-	-	-
Current treatment, No. (%)	36 (56.3%)	0 (0.0%)		
Psychotherapy, No. (%)	17 (26.6%)	0 (0.0%)	-	-
Antidepressants, No. (%)	26 (40.6%)	0 (0.0%)	-	-

a Education, categorized based on the highest level attained, was binarized for analyses (higher vocational education or university, yes/no).

### 3.2 Subjective stress responses

**Fig. 1:**
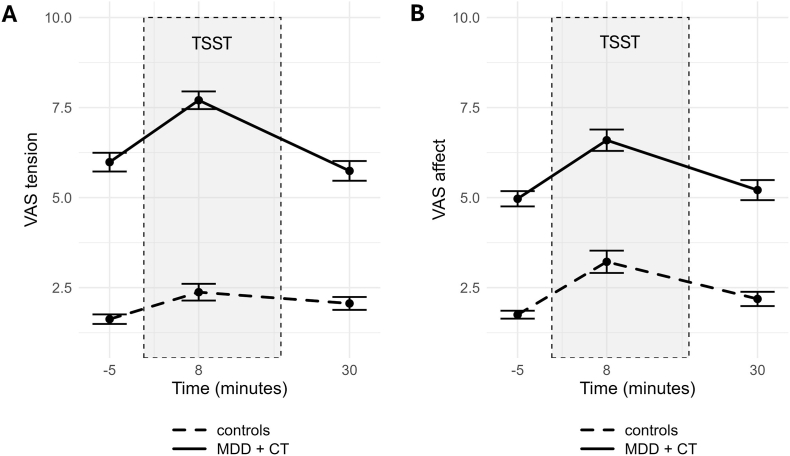
**Mean Visual Analogue Scale (VAS) scores before, during, and after the TSST by group. (A) Tension-related VAS scores**. Participants rated their current tension level from 1 ("Not tense at all") to 10 ("Very tense"). **(B) Affect-related VAS scores**. Participants rated their current mood from 1 ("Positive") to 10 ("Negative"). Ratings were collected at −5, +8, and +30 min relative to the onset of the TSST, with timepoint 0 indicating the start of the stressor. The shaded area (0-15 min) represents the TSST period. Mean scores ± standard error are displayed for each group. Solid lines indicate participants with MDD and a history of CT, dashed lines represent the control group. *Abbreviations:* VAS = Visual Analogue Scale; MDD = major depressive disorder; CT = childhood trauma; TSST = Trier Social Stress Test.

The stress induction paradigm elicited a significant subjective stress response in both groups, as indicated by significant increases in negative affect (MDD with CT: Δ 1.94, *t* = −7.63, *p*_*fdr*_ < 0.001; controls: Δ 1.69, t = −5.98, p_*fdr*_ < 0.001) and tension (MDD with CT: Δ 1.91, *t* = −7.51, *p*_*fdr*_ < 0.001; controls: Δ 0.97, *t* = −5.02, *p*_*fdr*_ < 0.001) from baseline to peak (Fig. 1). The MDD with CT group showed a greater increase in tension from baseline to peak than controls (*t* = 2.94, *p*_*fdr*_ < 0.01), whereas the increase in negative affect from baseline to peak did not differ significantly between groups (*t* = 0.66, *p*_*fdr*_ = 0.51). Analysis of AUC measures revealed that, for tension, individuals with MDD and CT had higher total VAS scores over time (AUCg = 236.38) than controls (AUCg = 74.81; *t* = 17.24, *p*_*fdr*_ < 0.001), while AUC relative to baseline did not differ significantly between groups (MDD with CT: AUCi = 28.64; controls: AUCi = 17.94; *t* = 1.28, *p*_*fdr*_ = 0.27). Similarly, for negative affect, individuals with MDD and CT showed higher total VAS scores over time (AUCg = 206.66) than controls (AUCg = 91.77; *t* = 11.03, *p*_*fdr*_ < 0.001), while VAS scores relative to baseline did not differ significantly between groups (MDD with CT: AUCi = 31.66; controls: AUCi = 30.52; *t* = 0.13, *p*_*fdr*_ = 0.90). VAS scores across all timepoints are shown in Supplementary Fig. 1.

### 3.3 fMRI network centrality

**Fig. 2:**
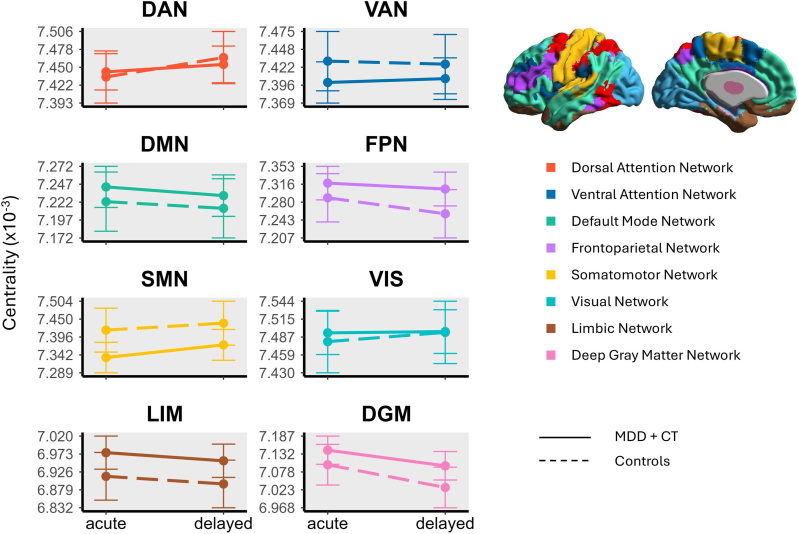
**Estimated eigenvector centrality across acute and delayed phases in major depressive disorder with childhood trauma (MDD + CT) and controls by brain network.** Estimated marginal means of eigenvector centrality per brain network derived from linear mixed-effects models adjusted for age (mean-centered) and sex. Results are shown per network for adults with major depressive disorder and childhood trauma (MDD + CT; solid lines) and healthy controls (dashed lines) during the acute (±15 min post-Trier Social Stress Test [TSST]) and delayed (±135 min post-TSST) phase. Brain networks include dorsal attention network (DAN; red), ventral attention network (VAN; dark blue), default mode network (DMN; green), frontoparietal network (FPN; purple), somatomotor network (SMN; yellow), visual network (VIS; light blue), limbic network (LIM; brown), and deep gray matter network (DGM; pink). Error bars represent 95% confidence intervals. No significant main effects of group or time, nor group × time interactions, were observed for any network. Brain network visualizations were generated using BrainNet Viewer (Fan et al., 2016).

Within-group linear mixed-effects models revealed no significant changes in EC between the acute and delayed stress phases for any network in either the MDD + CT group or the control group (all *p*_*fdr*_ ≥ 0.23; Table 2). Furthermore, no significant main effects of group or time, nor any group × time interactions were observed for EC in any network (all *p*_*fdr*_ ≥ 0.28 see Fig. 2,Table 3). Sensitivity analyses restricting the MDD + CT group to subgroups with severe MDD (IDS-SR score ≥39) or with two or more CT subtypes also revealed no significant effects of group, time, or their interaction on EC across networks (all *p*_*fdr*_ ≥ 0.22; Supplementary Tables 1 and 2, respectively).

**Table 2:** Network eigenvector centrality in individuals with MDD + CT and controls during the acute and delayed phase following stress induction.

Brain network	MDD + CT (N = 64)			Controls (N = 33)		
	Centrality (Mean ± SD) × 10^−3^		Mixed Effects Model	Centrality (Mean ± SD) × 10^−3^		Mixed Effects Model
	Acute	Delayed	Time effects	Acute	Delayed	Time effects
*VIS*	7.49 ± 0.12	7.49 ± 0.12	*t* = 0.11 *p*_*fdr*_ = 0.91	7.48 ± 0.14	7.50 ± 0.17	*t* = 0.49 *p*_*fdr*_ = 0.75
*SMN*	7.34 ± 0.13	7.37 ± 0.19	*t* = 1.38 *p*_*fdr*_ = 0.64	7.40 ± 0.21	7.43 ± 0.20	*t =* 0.49 *p*_*fdr*_ = 0.75
*DAN*	7.45 ± 0.11	7.46 ± 0.12	*t* = 0.61 *p*_*fdr*_ = 0.72	7.42 ± 0.10	7.46 ± 0.13	*t* = 1.10 *p*_*fdr*_ = 0.74
*VAN*	7.40 ± 0.10	7.40 ± 0.13	*t* = 0.33 *p*_*fdr*_ = 0.85	7.42 ± 0.14	7.42 ± 0.12	*t* = −0.17 *p*_*fdr*_ = 0.86
*Limbic*	6.96 ± 0.18	6.94 ± 0.19	*t* = −1.19 *p*_*fdr*_ = 0.64	6.95 ± 0.15	6.92 ± 0.15	*t* = −0.71 *p*_*fdr*_ = 0.75
*FPN*	7.33 ± 0.12	7.32 ± 0.13	*t* = −0.70 *p*_*fdr*_ = 0.72	7.28 ± 0.16	7.24 ± 0.13	*t* = −1.49 *p*_*fdr*_ = 0.58
*DMN*	7.24 ± 0.12	7.23 ± 0.11	*t* = −0.79 *p*_*fdr*_ = 0.72	7.23 ± 0.12	7.22 ± 0.11	*t* = −0.45 *p*_*fdr*_ = 0.75
*DGM*	7.14 ± 0.15	7.10 ± 0.17	*t* = −2.24 *p*_*fdr*_ = 0.23	7.11 ± 0.17	7.03 ± 0.21	*t* = −1.89 *p*_*fdr*_ = 0.54

Mean eigenvector centrality values ( × 10^−3^) and standard deviations are reported for each network during the acute (±15 min) and delayed (±135 min) phases following the TSST. Linear mixed-effects models were used to assess within-group changes in eigenvector centrality over time, with phase (acute vs. delayed) as the main predictor and age (mean-centered) and sex as covariates. Models were run separately for each network and each group (MDD + CT and controls). No significant time effects were observed in any network within either group. *Abbreviations:* VIS = Visual Network; SMN = Sensorimotor Network; DAN = Dorsal Attention Network; VAN = Ventral Attention Network; Limbic = Limbic Network; FPN = Frontoparietal Network; DMN = Default Mode Network; DGM = Deep Gray Matter Network; MDD = major depressive disorder; CT = childhood trauma, TSST = Trier Social Stress Test.

**Table 3:** Fixed effects from linear mixed-effects models predicting network eigenvector centrality from group, time, and their interaction.

Network	Predictor	df	Estimate (β)	SE	*F*-value	*p*_*fdr*_
VIS	Group	1, 93.47	1.41 × 10^−5^	3.18 × 10^−5^	0.08	0.76
	Time	1, 94.68	1.46 × 10^−5^	2.67 × 10^−5^	0.26	0.76
	Group × Time	1, 94.68	−1.27 × 10^−5^	3.26 × 10^−5^	0.15	0.97
SMN	Group	1, 93.53	−8.22 × 10^−5^	4.23 × 10^−5^	4.58	0.43
	Time	1, 94.88	2.08 × 10^−5^	4.06 × 10^−5^	1.41	0.67
	Group × Time	1, 94.88	1.71 × 10^−5^	4.96 × 10^−5^	0.12	0.97
DAN	Group	1, 93.34	2.63 × 10^−5^	2.63 × 10^−5^	0.00	0.76
	Time	1, 94.78	2.71 × 10^−5^	2.71 × 10^−5^	1.62	0.70
	Group × Time	1, 94.78	3.31 × 10^−5^	3.31 × 10^−5^	0.33	0.97
VAN	Group	1, 92.68	2.81 × 10^−5^	2.81 × 10^−5^	1.22	0.54
	Time	1, 93.94	2.48 × 10^−5^	2.48 × 10^−5^	0.00	0.86
	Group × Time	1, 93.94	3.04 × 10^−5^	3.04 × 10^−5^	0.11	0.97
LIM	Group	1, 93.42	6.23 × 10^−5^	4.04 × 10^−5^	2.76	0.51
	Time	1, 94.51	−1.99 × 10^−5^	2.65 × 10^−5^	1.62	0.76
	Group × Time	1, 94.51	−1.45 × 10^−6^	3.25 × 10^−5^	0.00	0.97
FPN	Group	1, 93.42	3.03 × 10^−5^	3.20 × 10^−5^	2.04	0.56
	Time	1, 94.57	−3.28 × 10^−5^	2.40 × 10^−5^	2.35	0.70
	Group × Time	1, 94.57	2.06 × 10^−5^	2.93 × 10^−5^	0.49	0.97
DMN	Group	1, 93.51	2.08 × 10^−5^	2.67 × 10^−5^	0.70	0.59
	Time	1, 94.70	−9.34 × 10^−6^	2.17 × 10^−5^	0.66	0.76
	Group × Time	1, 94.70	−2.94 × 10^−6^	2.65 × 10^−5^	0.01	0.97
DGM	Group	1, 92.66	4.45 × 10^−5^	4.01 × 10^−5^	2.53	0.54
	Time	1, 93.86	−6.97 × 10^−5^	3.25 × 10^−5^	8.69	0.28
	Group × Time	1, 93.86	2.20 × 10^−5^	3.98 × 10^−5^	0.30	0.97

Linear mixed-effects models were fitted separately for each brain network to examine the effects of group (MDD + CT vs. controls), time (acute vs. delayed phase), and their interaction on eigenvector centrality values. Models included age (mean-centered) and sex as covariates and a random intercept for subject. Reported are the estimated coefficients (β), standard errors (SE), F-values, degrees of freedom (df), and FDR-corrected *p*-values for each fixed effect. No significant group × time interaction was observed in any network. *Abbreviations:* VIS = Visual Network; SMN = Sensorimotor Network; DAN = Dorsal Attention Network; VAN = Ventral Attention Network; LIM = Limbic Network; FPN = Frontoparietal Network; DMN = Default Mode Network; DGM = Deep Gray Matter Network; MDD = major depressive disorder; CT = childhood trauma.

Consistent with this, no significant changes in DC across time were observed for any network within either group (all *p*_*fdr*_ ≥ 0.10; Supplementary Table 3). However, DC was significantly lower in the MDD + CT group compared to controls across all eight networks (VIS: β = −6.32, SE = 2.79, *p*_*fdr*_ < 0.05; SMN: β = −7.90, SE = 3.06, *p*_*fdr*_ < 0.05; DAN: β = −6.44, SE = 2.86, *p*_*fdr*_ < 0.05; VAN: β = −7.01, SE = 2.97, *p*_*fdr*_ < 0.05; LIM: β = −5.18, SE = 2.05, *p*_*fdr*_ < 0.05; FPN: β = −5.83, SE = 2.70, *p*_*fdr*_ < 0.05; DMN: β = −5.96, SE = 2.63, *p*_*fdr*_ < 0.05; DGM: β = −5.32, SE = 2.44, *p*_*fdr*_ < 0.05; Supplementary Table 4, Supplementary Fig. 2). Across groups, no significant differences in DC between acute and delayed stress phases were observed, nor were any group × time interaction effects (all *p*_*fdr*_ ≥ 0.07).

## 4 Discussion

This study investigated whether individuals with MDD and CT show altered resting-state network centrality during acute and delayed phases following psychosocial stress compared to controls. Both groups demonstrated a significant subjective stress response, as shown by increased levels of tension and negative affect during the TSST. Additionally, individuals with MDD and CT showed significantly greater overall tension and negative affect, as well as greater increases in tension than controls. However, looking at global centrality of eight functional brain networks, we observed no significant changes in EC from the acute to the delayed phase within either group. In addition, we found no group differences across stress phases, nor any group-specific differences over time. These findings held when restricting the MDD + CT group to individuals with more severe depression or multiple types of CT. Consistently, we found no changes in DC from the acute to the delayed stress phase within either group, nor any group-specific differences over time. However, the MDD + CT group presented lower overall DC compared to controls across all eight networks.

Contrary to our hypothesis, no differences in EC were observed between individuals with MDD and CT and controls following stress induction. This suggests that large-scale network centrality may not be the primary locus of stress-related alterations in this group. Notably, group differences did emerge in subjective tension responses. This aligns with previous findings indicating heightened self-reported life stress and greater daily stress reactivity among individuals with MDD (Maquet et al., 2020; van et al., 2015; Pasteuning et al., 2025b; Van der Stouwe et al., 2019) and those with a history of CT (Wang et al., 2025; Mayer et al., 2022). Importantly, our null findings in EC do not imply that resting-state alterations are absent in individuals with MDD and CT altogether. Prior studies have reported differences in intrinsic functional connectivity in both MDD and CT in the absence of stress: individuals with CT show alterations primarily in the fronto-limbic circuit and DMN (Ross et al., 2021; Holz et al., 2023), while in MDD, altered functional connectivity has been observed within the DMN and FPN, as well as between the DMN and FPN, the DMN and SMN, the LIM and DMN, the LIM and FPN, and the VAN and DAN (Zhang et al., 2025). Consistent with these widespread network alterations, our findings show lower DC in individuals with MDD and CT compared to controls across acute and delayed stress phases for all eight networks, which may reflect a difference in global brain connectivity unrelated to stress induction.

The lack of differences in network centrality between acute and delayed stress phases across groups was not in line with previous studies suggesting significant changes in the VAN and the DAN following stress (Van Oort, 2017; Broeders et al., 2021). This discrepancy may be related to our outcome measures, as global connectivity measures alone may not capture the full complexity of brain network organization. For instance, EC and DC capture temporally averaged and spatially distributed connectivity patterns, making them less sensitive to subtle, transient, or regionally localized reconfigurations of functional connectivity (Zuo et al., 2012). Additionally, while recent research has suggested moderate-to-good test-retest reliability for DC, EC appeared more sensitive to multi-session variability, although sample characteristics may play a role (Mullin et al., 2025). Therefore, other, more targeted measures of functional brain connectivity may yield different findings. For instance, dynamic functional connectivity allows for examining network dynamics on a smaller temporal scale, providing more insight into how the brain adapts to environmental stimuli (Cohen, 2018). Consequently, dynamic functional connectivity may be more sensitive to subtle and rapid changes in network interactions, which may be particularly relevant in stress designs.

Alternatively, the current findings may indicate that stress vulnerability following CT manifests more prominently in subjective and behavioral outcomes. For instance, both MDD and CT have been consistently associated with functional disturbances in emotion regulation and cognitive processing (Dotson et al., 2020; Gruhn et al., 2020; Masson et al., 2016; Nagy et al., 2021; Visted et al., 2018; Wang et al., 2024), which can both be disrupted under stress (Langer et al., 2025; Shields et al., 2016; Wessa et al., 2024). Therefore, functional brain connectivity during cognitive and emotional processing may be better suited to examine stress-related differences in neural mechanisms following CT. This ties into a more general point that CT needs to be considered within its broader, multimodal context. CT is associated with a complex variety of alterations across multiple domains, including molecular, neurobiological, psychosocial and behavioral mechanisms, which are closely interconnected (Pasteuning et al., 2025a). Employing a more comprehensive approach and examining these modalities in parallel, both in the presence and absence of stress, is paramount to determine at which level group differences are most pronounced and thereby identify the most promising targets for further investigation and treatment. Based on the present findings, it may be more valuable to explore beyond stress-induced resting-state changes and examine other modalities, such as subjective and behavioral responses.

A key strength of this study is that it is the first to examine changes in functional brain networks related to stress processing in individuals with MDD and CT. In addition, we employed an extensive, multi-session fMRI paradigm following stress induction in a relatively large sample of patients with MDD, who reported high symptom severity (mean IDS-SR = 39.4; scores ≥39 indicate severe depression). Yet, several limitations should be noted. First, the lack of a baseline fMRI scan prior to stress induction limits the ability to determine the extent of stress-induced alterations within and between individuals. As a result, it remains difficult to evaluate whether the absence of differences in network centrality between acute and delayed stress phases reflects a true lack of stress-related effects. Even though previous research showed no significant differences in network centrality between resting-state scans obtained before and after stress induction, this limitation remains (Broeders et al., 2021). Second, the absence of cortisol measurements precluded the possibility of differentiating between cortisol responders and non-responders, which may be relevant given that responder status can influence neural trajectories following stress. For example, Vaisvaser et al. (2013) demonstrated that both responders and non-responders showed increased amygdala-hippocampus resting-state connectivity immediately after stress induction, whereas only non-responders exhibited sustained increases 2 h later (Vaisvaser et al., 2013). Finally, the MDD + CT and control groups differed significantly in age and sex. Therefore, we corrected for these variables in mixed model analyses. However, controlling for covariates that bear a strong association with group membership raises the possibility of reducing or obscuring genuine group effects, especially as age and sex have been linked to differences in neurobiological characteristics of MDD including functional resting-state alterations (Hu et al., 2022; Tian et al., 2024; He et al., 2022; Schisterman et al., 2009). Ideally, subgroup analyses stratified by age or sex would clarify such patterns, but the current sample (size) did not permit this approach.

Building on these findings, we propose several recommendations for future research. First, it is recommended that future research incorporates active fMRI paradigms (e.g., tasks or movie-watching) in the context of stress to provide a more comprehensive picture of neural stress processing. Specifically, given the consistent associations between MDD, CT, and stress to both emotion regulation and cognitive functioning, tasks probing these domains under stress could reveal group differences that remain undetected in global resting-state network centrality measures alone. Second, as the present study sought to offer a preliminary investigation of neural stress responses in a high-burden group, only patients with both MDD and CT were included. However, previous research has reported distinct, cumulative, and interactive alterations in resting-state network connectivity related to MDD and CT (Luo et al., 2022; Zou et al., 2024; Xia et al., 2024). To disentangle MDD- and CT-specific effects and facilitate better comparison of findings across studies, we recommend researchers employ full factorial designs with four groups (MDD only, CT only, both, and neither). Third, the impact of measurement timing on observed recovery effects remains unclear, as most studies to date have focused on acute stress effects, while few included delayed effects (>30 min post-stressor) (Van et al., 2017b; Broeders et al., 2021). Given the temporal specificity of stress effects on brain connectivity, differences in fMRI measurement timing can substantially shape the results (Hermans et al., 2014). Therefore, we encourage future studies to incorporate measurements spanning longer time frames to more accurately map delayed stress effects. Finally, we recommend following guidelines to support methodological rigor and participant well-being in stress-induction studies involving clinical populations (Broeder et al., 2025).

In conclusion, the present study provides an initial examination of neural stress-response trajectories in individuals with MDD and CT using a whole-brain, network-based approach. No stress-related changes in large-scale resting-state network centrality were observed, suggesting that this global measure may not be a sensitive indicator of stress-related vulnerability in MDD and CT. Despite null findings in EC, subjective stress responses did distinguish groups, underscoring the value of studying CT within a broader, multimodal context. Future studies that investigate brain network changes associated with processing emotional and cognitively demanding stimuli after stress induction, employ different, more targeted measures of functional brain connectivity, and incorporate biological stress markers may more precisely map the pathways by which MDD and CT shape stress reactivity and recovery.

## Funding

This work was supported by 10.13039/501100001826ZonMw (grant number 09150171910042) and the Dutch 10.13039/501100000942Brain Foundation (Hersenstichting; DR-2019-00290).

## CRediT authorship contribution statement

**C. Broeder:** Conceptualization, Formal analysis, Investigation, Methodology, Project administration, Visualization, Writing – original draft, Writing – review & editing. **J.M. Pasteuning:** Conceptualization, Formal analysis, Investigation, Methodology, Project administration, Visualization, Writing – original draft, Writing – review & editing. **T.A.A. Broeders:** Methodology, Resources, Writing – review & editing. **F. Linsen:** Conceptualization, Investigation, Methodology, Project administration, Writing – review & editing. **M.M. Schoonheim:** Conceptualization, Methodology, Supervision, Writing – review & editing. **M.S.C. Sep:** Conceptualization, Methodology, Supervision, Writing – review & editing. **C.H. Vinkers:** Conceptualization, Funding acquisition, Methodology, Supervision, Writing – review & editing.

## Declaration of competing interest

The authors declare that they have no known competing financial interests or personal relationships that could have appeared to influence the work reported in this paper.

## Data Availability

Data will be made available on request.
