# Supplementary material for: Brain network centrality following stress in adults with major depressive disorder and childhood trauma
Source: Neurobiol Stress. 2026 Apr 12;42:100817. doi: 10.1016/j.ynstr.2026.100817 (PMC13125892; doi:10.1016/j.ynstr.2026.100817)
Supplement: Multimedia component 1 [file mmc1.docx]

**Supplementary Material**


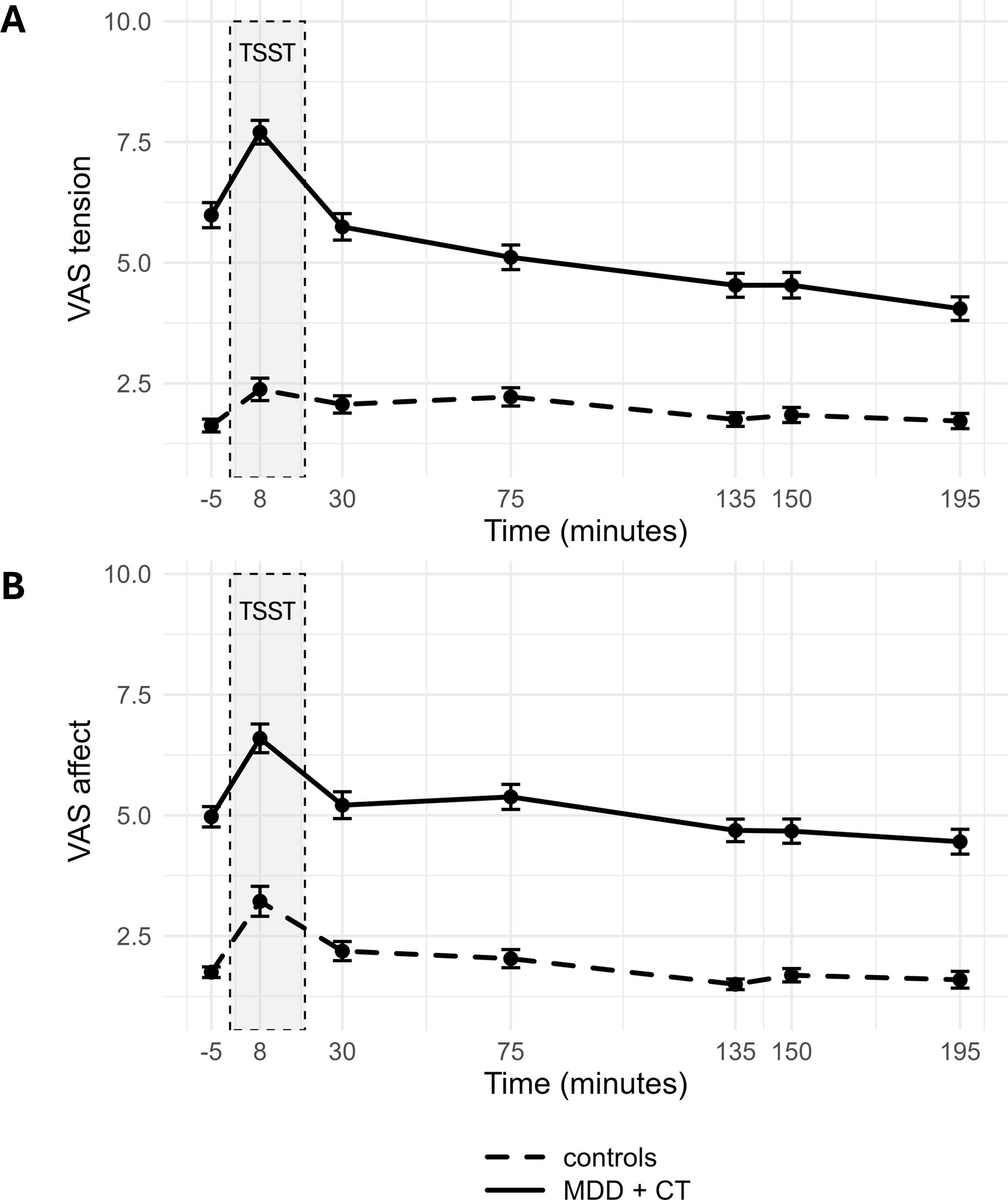


**Supplementary Figure 1. Mean Visual Analogue Scale (VAS) scores across all timepoints by group.
(A)** **Tension-related VAS scores**. Participants rated their subjective tension from 1 ("Not tense at all") to 10 ("Very tense"). **(B)** **Affect-related VAS scores**. Participants rated their mood from 1 ("Positive") to 10 ("Negative"). Ratings were collected at -5, +8, +30, +75, +135, +150, and +195 minutes relative to the onset of the Trier Social Stress Test (TSST), with time 0 indicating the start of the stressor and the shaded area (0–15 minutes) representing the TSST period. Mean scores ± standard error are displayed for each group. Solid lines indicate participants with major depressive disorder (MDD) and a history of childhood trauma (CT), dashed lines represent the control group.

*Abbreviations:* VAS = Visual Analogue Scale; MDD = major depressive disorder; CT = childhood trauma; TSST = Trier Social Stress Test.

**Supplementary Table 1. Fixed effects from linear mixed-effects models predicting network eigenvector centrality from group, time, and their interaction in individuals with severe major depressive disorder (IDS-SR score ≥ 39) with childhood trauma and controls.**

| **Network** | **Predictor** | **df** | **Estimate (β)** | **SE** | ***F*-value** | ***p_fdr_*** |
| --- | --- | --- | --- | --- | --- | --- |
| VIS | Group | 1, 65.00 | 3.37×10^⁻5^ | 3.83×10^⁻5^ | 0.46 | 0.61 |
|  | Time | 1, 67.00 | 1.46×10^⁻5^ | 2.86×10^⁻5^ | 0.03 | 0.77 |
|  | Group × Time | 1, 67.00 | -2.26×10^⁻5^ | 3.90×10^⁻5^ | 0.34 | 0.96 |
| SMN | Group | 1, 65.00 | -9.28×10^⁻5^ | 5.21×10^⁻5^ | 3.90 | 0.55 |
|  | Time | 1, 67.00 | 2.08×10^⁻5^ | 4.40×10^⁻5^ | 0.98 | 0.77 |
|  | Group × Time | 1, 67.00 | 1.77×10^⁻5^ | 6.00×10^⁻5^ | 0.09 | 0.96 |
| DAN | Group | 1, 65.00 | 8.95×10^⁻7^ | 2.89×10^⁻5^ | 0.03 | 0.98 |
|  | Time | 1, 67.00 | 3.06×10^⁻5^ | 2.66×10^⁻5^ | 1.99 | 0.68 |
|  | Group × Time | 1, 67.00 | -9.95×10^⁻6^ | 3.63×10^⁻5^ | 0.08 | 0.96 |
| VAN | Group | 1, 65.00 | -3.57×10^⁻5^ | 3.25×10^⁻5^ | 1.15 | 0.55 |
|  | Time | 1, 67.00 | -4.47×10^⁻6^ | 2.40×10^⁻5^ | 0.01 | 0.85 |
|  | Group × Time | 1, 67.00 | 1.13×10^⁻5^ | 3.28×10^⁻5^ | 0.12 | 0.96 |
| LIM | Group | 1, 65.00 | 3.46×10^⁻5^ | 4.77×10^⁻5^ | 0.55 | 0.63 |
|  | Time | 1, 67.00 | -1.99×10^⁻5^ | 2.88×10^⁻5^ | 1.28 | 0.77 |
|  | Group × Time | 1, 67.00 | -4.71×10^⁻6^ | 3.94×10^⁻5^ | 0.01 | 0.96 |
| FPN | Group | 1, 65.00 | 2.07×10^⁻5^ | 3.81×10^⁻5^ | 1.37 | 0.67 |
|  | Time | 1, 67.00 | -3.28×10^⁻5^ | 2.47×10^⁻5^ | 0.64 | 0.68 |
|  | Group × Time | 1, 67.00 | 3.848×10^⁻5^ | 3.38×10^⁻5^ | 1.30 | 0.96 |
| DMN | Group | 1, 65.00 | 3.58×10^⁻5^ | 2.98×10^⁻5^ | 1.87 | 0.55 |
|  | Time | 1, 67.00 | -9.34×10^⁻6^ | 2.21×10^⁻5^ | 0.44 | 0.77 |
|  | Group × Time | 1, 67.00 | -1.39×10^⁻6^ | 3.02×10^⁻5^ | 0.00 | 0.96 |
| DGN | Group | 1, 65.00 | 6.13×10^⁻5^ | 4.70×10^⁻5^ | 2.18 | 0.55 |
|  | Time | 1, 67.00 | -6.97×10^⁻5^ | 3.49×10^⁻5^ | 8.92 | 0.40 |
|  | Group × Time | 1, 67.00 | -2.97×10^⁻5^ | 4.76×10^⁻5^ | 0.00 | 0.96 |

Linear mixed-effects models were fitted separately for each brain network to examine the effects of group (MDD + CT vs. controls), phase (acute vs. delayed), and their interaction on eigenvector centrality values. Models included centered age and sex as covariates and a random intercept for subject. Reported are the estimated coefficients (β), standard errors (SE), F-values, degrees of freedom (df), and p-values for each fixed effect.

*Abbreviations:* VIS = Visual Network; SMN = Sensorimotor Network; DAN = Dorsal Attention Network; VAN = Ventral Attention Network; LIM = Limbic Network; FPN = Frontoparietal Network; DMN = Default Mode Network; DGM = Deep Gray Matter Network; MDD = major depressive disorder; CT = childhood trauma.

**Supplementary Table 2. Fixed effects from linear mixed-effects models predicting network eigenvector centrality from group, time, and their interaction in individuals with major depressive disorder with ≥2 childhood trauma subtypes and controls.**

| **Network** | **Predictor** | **df** | **Estimate (β)** | **SE** | ***F*-value** | ***p_fdr_*** |
| --- | --- | --- | --- | --- | --- | --- |
| VIS | Group | 1, 80.57 | 1.23×10^⁻5^ | 3.39×10^⁻5^ | 0.05 | 0.79 |
|  | Time | 1, 81.74 | 1.46×10^⁻5^ | 2.69×10^⁻5^ | 0.27 | 0.77 |
|  | Group × Time | 1, 81.74 | -1.13×10^⁻5^ | 3.43×10^⁻5^ | 0.11 | 0.92 |
| SMN | Group | 1, 80.64 | -7.54×10^⁻5^ | 4.11×10^⁻5^ | 3.09 | 0.46 |
|  | Time | 1, 81.95 | 2.08×10^⁻5^ | 4.53×10^⁻5^ | 1.40 | 0.77 |
|  | Group × Time | 1, 81.95 | 2.21×10^⁻5^ | 5.39×10^⁻5^ | 0.17 | 0.92 |
| DAN | Group | 1, 163.00 | 2.01×10^⁻5^ | 2.62×10^⁻5^ | 0.56 | 0.79 |
|  | Time | 1, 163.00 | 3.06×10^⁻5^ | 2.70×10^⁻5^ | 2.21 | 0.71 |
|  | Group × Time | 1, 163.00 | -1.02×10^⁻5^ | 3.43×10^⁻5^ | 0.09 | 0.92 |
| VAN | Group | 1, 79.84 | -3.14×10^⁻5^ | 3.04×10^⁻5^ | 0.70 | 0.62 |
|  | Time | 1, 81.04 | -4.47×10^⁻5^ | 2.53×10^⁻5^ | 0.10 | 0.86 |
|  | Group × Time | 1, 81.04 | 1.93×10^⁻5^ | 3.23×10^⁻5^ | 0.36 | 0.92 |
| LIM | Group | 1, 80.47 | 8.03×10^⁻5^ | 4.11×10^⁻5^ | 4.04 | 0.45 |
|  | Time | 1, 81.52 | -1.99×10^⁻5^ | 2.53×10^⁻5^ | 2.24 | 0.77 |
|  | Group × Time | 1, 81.52 | -8.43×10^⁻6^ | 3.23×10^⁻5^ | 0.07 | 0.92 |
| FPN | Group | 1, 80.48 | 3.35×10^⁻5^ | 3.31×10^⁻5^ | 1.58 | 0.62 |
|  | Time | 1, 81.57 | -3.28×10^⁻5^ | 2.30×10^⁻5^ | 3.85 | 0.62 |
|  | Group × Time | 1, 81.57 | 8.10×10^⁻5^ | 2.93×10^⁻5^ | 0.08 | 0.92 |
| DMN | Group | 1, 80.62 | 3.74×10^⁻6^ | 2.72×10^⁻5^ | 0.01 | 0.79 |
|  | Time | 1, 81.80 | -9.34×10^⁻6^ | 2.20×10^⁻5^ | 1.26 | 0.77 |
|  | Group × Time | 1, 81.80 | -1.27×10^⁻5^ | 2.78×10^⁻5^ | 0.21 | 0.92 |
| DGM | Group | 1, 80.86 | 3.63×10^⁻5^ | 4.18×10^⁻5^ | 1.78 | 0.62 |
|  | Time | 1, 81.97 | -6.97×10^⁻5^ | 3.11×10^⁻5^ | 8.26 | 0.22 |
|  | Group × Time | 1, 81.97 | 2.60×10^⁻5^ | 3.94×10^⁻5^ | 0.44 | 0.92 |

Linear mixed-effects models were fitted separately for each brain network to examine the effects of group (MDD + CT vs. controls), phase (acute vs. delayed), and their interaction on eigenvector centrality values. Models included centered age and sex as covariates and a random intercept for subject. Reported are the estimated coefficients (β), standard errors (SE), F-values, degrees of freedom (df), and p-values for each fixed effect.

*Abbreviations:* VIS = Visual Network; SMN = Sensorimotor Network; DAN = Dorsal Attention Network; VAN = Ventral Attention Network; LIM = Limbic Network; FPN = Frontoparietal Network; DMN = Default Mode Network; DGM = Deep Gray Matter Network; MDD = major depressive disorder; CT = childhood trauma.

**Supplementary Table 3. Network degree centrality in individuals with major depressive disorder with childhood trauma and controls during acute and delayed phases following stress induction.**

|  | | **MDD + CT** (*N* = 64) | | | | |  | **Controls** (*N* = 33) | | | | |  |
| --- | --- | --- | --- | --- | --- | --- | --- | --- | --- | --- | --- | --- | --- |
|  | | DC (Mean ± SD) | | |  | Mixed Effects Model |  | DC (Mean ± SD) | | |  | Mixed Effects Model | |
| **Brain network** | | **Acute** |  | **Delayed** |  | **Time effects** |  | **Acute** |  | **Delayed** |  | **Time effects** | |
|  | *VIS* | 23.41 ±  11.91 |  | 25.45 ±  11.49 |  | *t* = 2.07  *p_fdr_* = 0.10 |  | 29.13 ±  13.37 |  | 31.00 ±  12.71 |  | *t* = 1.30  *p_fdr_* = 0.54 | |
|  | *SMN* | 20.55 ±  12.47 |  | 23.32 ±  12.75 |  | *t* = 2.21  *p_fdr_* = 0.10 |  | 27.66 ±  14.70 |  | 29.88 ±  14.79 |  | *t* = 1.33  *p_fdr_* = 0.54 | |
|  | *DAN* | 22.46 ±  12.17 |  | 24.69 ±  11.95 |  | *t* = 2.04  *p_fdr_* = 0.10 |  | 28.00 ±  13.87 |  | 30.19 ±  12.99 |  | *t* = 1.52  *p_fdr_* = 0.54 | |
|  | *VAN* | 21.35 ±  12.12 |  | 23.56 ±  12.39 |  | *t* = 1.30  *p_fdr_* = 0.10 |  | 27.88 ±  14.78 |  | 29.41 ±  13.91 |  | *t* = 0.94  *p_fdr_* = 0.54 | |
|  | *LIM* | 13.89 ±  7.90 |  | 15.31 ±  7.93 |  | *t* = 1.74  *p_fdr_* = 0.10 |  | 19.09 ±  11.09 |  | 20.10 ±  10.11 |  | *t* = 0.84  *p_fdr_* = 0.54 | |
|  | *FPN* | 20.33 ±  11.71 |  | 21.94 ±  10.58 |  | *t* = 1.74  *p_fdr_* = 0.10 |  | 25.11 ±  13.45 |  | 25.89 ±  12.25 |  | *t* = 0.58  *p_fdr_* = 0.64 | |
|  | *DMN* | 18.80 ±  10.81 |  | 20.50 ±  10.73 |  | *t* = 1.82  *p_fdr_* = 0.10 |  | 24.20 ±  12.71 |  | 25.58 ±  12.25 |  | *t* = 1.09  *p_fdr_* = 0.54 | |
|  | *DGM* | 16.55 ±  10.18 |  | 17.72 ±  8.87 |  | *t* = 1.38  *p_fdr_* = 0.20 |  | 21.47 ±  12.56 |  | 21.83 ±  11.95 |  | *t* = 0.26  *p_fdr_* = 0.80 | |

Mean degree centrality (DC) values and standard deviations are reported for each network during the acute (± 15 min) and delayed (± 135 min) phases following the onset of the Trier Social Stress Test. Linear mixed-effects models were used to assess within-group changes in DC over time, with phase (acute, delayed) as the main predictor and age (mean-centered) and sex as covariates. Models were run separately for each network and each group (MDD + CT and controls).

*Abbreviations:* VIS = Visual Network; SMN = Sensorimotor Network; DAN = Dorsal Attention Network; VAN = Ventral Attention Network; Limbic = Limbic Network; FPN = Frontoparietal Network; DMN = Default Mode Network; DGM = Deep Gray Matter Network; MDD = major depressive disorder; CT = childhood trauma.

**Supplementary Table 4. Fixed effects from linear mixed-effects models predicting network degree centrality from group, time, and their interaction.**

| **Network** | **Predictor** | **df** | **Estimate (β)** | **SE** | ***F*-value** | ***p_fdr_*** |
| --- | --- | --- | --- | --- | --- | --- |
| VIS | Group | 1, 94.27 | -6.32 | 2.79 | 5.45 | **<0.05*** |
|  | Time | 1, 95.32 | 1.86 | 1.42 | 5.13 | 0.07 |
|  | Group × Time | 1, 95.32 | 0.22 | 1.74 | 0.02 | 0.97 |
| SMN | Group | 1, 94.32 | -7.90 | 3.06 | 7.06 | **<0.05*** |
|  | Time | 1, 95.39 | 2.22 | 1.73 | 5.51 | 0.07 |
|  | Group × Time | 1, 95.39 | 0.57 | 2.13 | 0.07 | 0.97 |
| DAN | Group | 1, 94.29 | -6.44 | 2.86 | 5.60 | **<0.05*** |
|  | Time | 1, 95.35 | 2.19 | 1.52 | 5.72 | 0.07 |
|  | Group × Time | 1, 95.35 | 0.08 | 1.87 | 0.00 | 0.97 |
| VAN | Group | 1, 94.29 | -7.01 | 2.97 | 5.60 | **<0.05*** |
|  | Time | 1, 95.35 | 1.52 | 1.59 | 3.76 | 0.09 |
|  | Group × Time | 1, 95.35 | 0.75 | 1.96 | 0.15 | 0.97 |
| LIM | Group | 1, 94.33 | -5.18 | 2.05 | 6.66 | **<0.05*** |
|  | Time | 1, 95.40 | 1.01 | 1.17 | 2.88 | 0.12 |
|  | Group × Time | 1, 95.40 | 0.44 | 1.44 | 0.09 | 0.97 |
| FPN | Group | 1, 94.26 | -5.83 | 2.70 | 4.41 | **<0.05*** |
|  | Time | 1, 95.31 | 0.79 | 1.33 | 2.20 | 0.16 |
|  | Group × Time | 1, 95.31 | 0.85 | 1.63 | 0.27 | 0.97 |
| DMN | Group | 1, 94.26 | -5.96 | 2.63 | 5.36 | **<0.05*** |
|  | Time | 1, 95.31 | 1.38 | 1.30 | 3.75 | 0.09 |
|  | Group × Time | 1, 95.31 | 0.34 | 1.60 | 0.05 | 0.97 |
| DGM | Group | 1, 94.25 | -5.32 | 2.44 | 4.55 | **<0.05*** |
|  | Time | 1, 95.30 | 0.36 | 1.28 | 0.91 | 0.34 |
|  | Group × Time | 1, 95.30 | 0.78 | 1.58 | 0.25 | 0.97 |

Linear mixed-effects models were fitted separately for each brain network to examine the effects of group (MDD + CT vs. controls), time (acute vs. delayed phase), and their interaction on degree centrality values. Models included age (mean-centered) and sex as covariates and a random intercept for subject. Reported are the estimated coefficients (β), standard errors (SE), F-values, degrees of freedom (df), and false discovery rate (FDR)-corrected *p*-values for each fixed effect. Asterisks indicate significant effects that survived FDR correction.

*Abbreviations:* VIS = Visual Network; SMN = Sensorimotor Network; DAN = Dorsal Attention Network; VAN = Ventral Attention Network; LIM = Limbic Network; FPN = Frontoparietal Network; DMN = Default Mode Network; DGM = Deep Gray Matter Network; MDD = major depressive disorder; CT = childhood trauma.


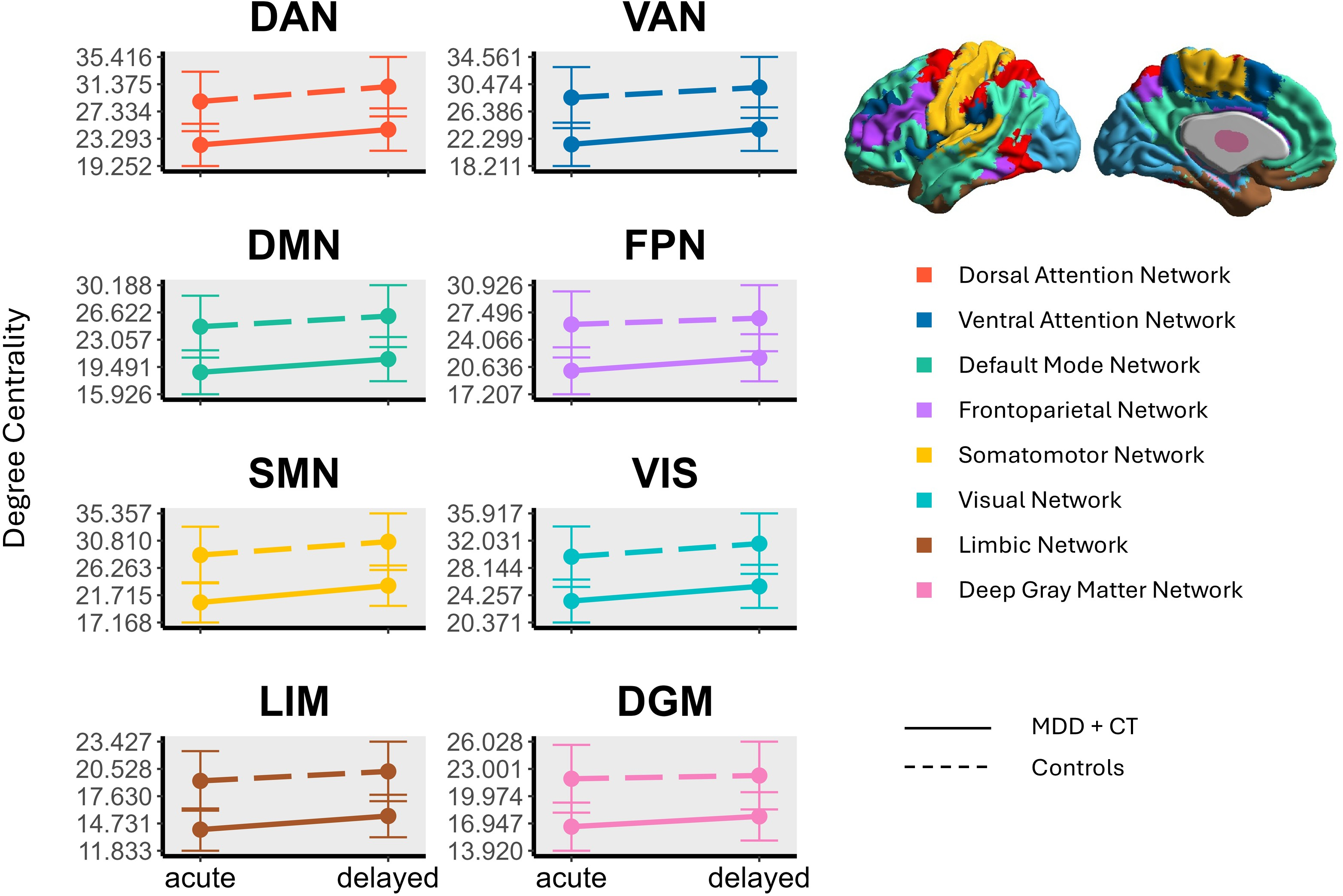


**Supplementary Figure 2. Estimated degree centrality across acute and delayed stress phases in individuals with major depressive disorder with childhood trauma (MDD + CT) and controls by brain network.**

Estimated marginal means of degree centrality per brain network derived from linear mixed-effects models adjusted for age (mean-centered) and sex. Results are shown per network for adults with major depressive disorder and childhood trauma (MDD + CT; solid lines) and healthy controls (dashed lines) during acute (± 15 minutes post-Trier Social Stress Test [TSST]) and delayed (± 135 minutes post-TSST) stress phases. Brain networks include dorsal attention network (DAN; red), ventral attention network (VAN; dark blue), default mode network (DMN; green), frontoparietal network (FPN; purple), somatomotor network (SMN; yellow), visual network (VIS; light blue), limbic network (LIM; brown), and deep gray matter network (DGM; pink). Error bars represent 95% confidence intervals. DC was significantly higher in the MDD + CT group compared to controls across all eight networks. Brain network visualizations were generated using BrainNet Viewer (Xia et al., 2013).

**References**

Xia, M., Wang, J., & He, Y. (2013). BrainNet Viewer: a network visualization tool for human brain connectomics. *PLoS One, 8*(7), e68910.
